# RSK-c-Fos in KSHV lytic progression

**DOI:** 10.18632/oncotarget.5262

**Published:** 2015-08-26

**Authors:** Xiaojuan Li, Ersheng Kuang

**Affiliations:** Institute of Human Virology, Zhongshan School of Medicine, Sun Yat-sen University, Guangzhou, China

**Keywords:** KSHV, lytic replication, RSK, c-Fos

Kaposi's sarcoma (KS) is a common malignancy in untreated AIDS patients that is caused by the infection of Kaposi's sarcoma-associated herpesvirus (KSHV). KS lesions are characterized by spindle-shaped endothelial cells with infiltration of inflammatory cells and neo-angiogenesis. KSHV commonly establishes default latent infection, but lytic replications occur in a small fraction of cells and play critical roles in KS pathogenesis by serving as a reservoir of infectious virion for persistent infection and inducing continuous paracrine factors for tumor progression. KSHV lytic infection triggers a variety of cellular machinery for replication and remodels cellular transcriptome. Distinct transcriptional patterns are exhibited in the immediate early (IE) and late stages of lytic replication. Previous studies have revealed that multiple pathways activate IE gene expression during lytic infection and reactivation from latency. However, the mechanism of late lytic gene expression regulation has remained largely undefined for a long time. Recently, studies have revealed that both host and viral factors, ORF45-prolonged c-Fos and TATA box binding protein (TBP)-like protein ORF24, are required for late lytic transcription [1, 2].

**Figure 1:**
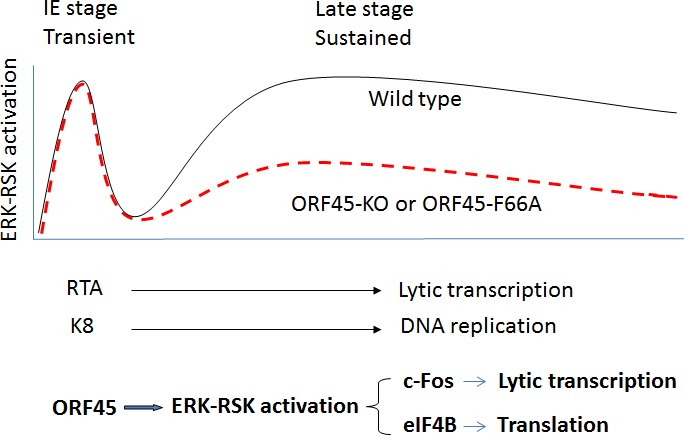
Sustained ERK-RSK activation and substrates during KSHV lytic replication Extracellular mitogen-like stimuli or glycoproteins of KSHV virion trigger transient ERK-RSK activation to induce expression of the IE genes RTA, K8 and ORF45 during the IE stage of primary infection or reactivation from latency. Consequently, ORF45 and other viral products trigger sustained ERK-RSK activation during the late stage; ORF45-null or ORF45-F66A mutagenesis dramatically reduce their activation. As results, sustained ERK-RSK activation phosphorylates and activates eIF4B and c-Fos to promote translation and lytic transcription during late stage of KSHV lytic life cycle, respectively.

Our previous observations demonstrated that KSHV tegument protein ORF45 interacts with p90 ribosomal S6 protein kinase (RSK) and induces reciprocal ERK-RSK activation through the formation of ORF45-ERK-RSK multiprotein complexes that protect active ERK-RSK from dephosphorylation, consequently activating a sustained ERK-MAPK cascade that is essential for lytic replication [3, 4]. Deficiency of sustained ERK-RSK activation by ORF45-null or ORF45-F66A point mutagenesis significantly reduces the late lytic gene expression and progeny virion yield [5], indicating the essential role of ORF45-mediated sustained ERK-RSK activation during late lytic replication. This sustained signaling phosphorylates eukaryotic translation initiation factor eIF4B and therefore facilitates translation and promotes virion production [6]. Our latest studies have further revealed that sustained activation of this pathway prolongs c-Fos accumulation and activation to accelerate viral lytic transcription during the late stage of lytic replication [1], providing the evidence that ORF45-mediated sustained ERK-RSK activation promotes viral lytic replication at both the transcription and translation levels (Figure 1).

As the sensor of sustained ERK-RSK activation, prolonged c-Fos activation accelerates late lytic transcription, whereas transient ERK-RSK activation-mediated rapid c-Fos activation induces IE gene expression. Two types of c-Fos exhibit the distinct promoter-binding profile in the KSHV DNA genome [1] and therefore activate different transcriptional patterns. Depletion of c-Fos expression reduces late lytic gene expression (even when expression of the IE genes RTA and ORF45 is primarily unaffected); ectopic expression of wild-type c-Fos increases lytic transcription, whereas phosphorylation site-mutated c-Fos exerts a dominant negative effect on the transcription of a panel of lytic genes. Thus, our studies demonstrated that c-Fos plays an important role in KSHV lytic transcriptional progression following ORF45-mediated sustained ERK-RSK activation.

γ2-herpesviruses encode conserved ORF45 homologues that share a RSK-binding region [5], and an ORF45 homologue of rhesus monkey rhadinovirus induces ERK and RSK activation [7], suggesting that ORF45 commonly induces sustained ERK-RSK activation during lytic replication of γ2-herpesvirus (*Rhadinovirus*) and that prolonged c-Fos regulates their lytic progression. However, the similar RSK-binding region is not found in ORF45 homologues of γ1-herpesvirus (*Lymphocryptovirus*), such as EBV BKRF4, suggesting this mechanism does not occur in other families of herpesviruses and that there are other conserved mechanisms for the lytic progression of these herpesviruses.

Our studies have shown that KSHV genes are divided into c-Fos-dependent and c-Fos-independent clusters that are activated by c-Fos directly or by secondary transactivation of RTA or other c-Fos targets, respectively. The expression of c-Fos-independent clusters still requires sustained ERK-RSK activation, suggesting that other transcription factors or cooperative viral and cellular partners are involved in KSHV viral transcription. One study has revealed that the transcription factor Elk-1 is activated by ERK-RSK signaling of the ORF45 homologue of γ2-herpesvirus [7]. Further systemic studies to identify the new targets of sustained ERK-RSK activation will provide the new insights into late lytic progression, including late transcription, replication, virion assembly and maturation, etc.

We have revealed the role of the ORF45-ERK-RSK cascade in viral lytic progression, while the functions in oncogenic transformation and host-virus interaction remain largely unknown. Prolonged c-Fos activation may attenuate inflammatory responses, which play important roles in tumorigenesis and the progression of KSHV-associated diseases. Furthermore, sustained ERK-RSK activation may be involved in the conversion of cell morphology and transformation. Given that RSK and other MAPK inhibitors significantly suppress KSHV lytic replication, it is interesting to evaluate the therapeutic role of ERK-RSK signaling in KSHV pathogenesis. Additionally, targeting sustained ERK-RSK activation is a promising therapeutic approach for KSHV-related diseases.

In conclusion, our studies revealed that KSHV ORF45 induces sustained ERK-MAPK activation during late lytic replication. Consequently, c-Fos accumulation and transactivation are prolonged to accelerate lytic transcription during the late stage of KSHV lytic life cycle. Rapid and prolonged c-Fos activation mediates lytic transcription during the IE and late stage of KSHV lytic replication, respectively, indicating that c-Fos timing determines KSHV lytic progression. The prolonged c-Fos activation may represent a common regulation for late lytic transcription and replication of γ2-herpesviruses.
